# Uterine Tumors Resembling Ovarian Sex Cord Tumors (UTROSCTs): A Scoping Review of 511 Cases, Including 2 New Cases

**DOI:** 10.3390/medicina60010179

**Published:** 2024-01-19

**Authors:** Rafał Watrowski, Mario Palumbo, Serena Guerra, Alessandra Gallo, Brunella Zizolfi, Pierluigi Giampaolino, Giuseppe Bifulco, Attilio Di Spiezio Sardo, Maria Chiara De Angelis

**Affiliations:** 1 Department of Obstetrics and Gynecology, Helios Hospital Müllheim, 79379 Müllheim, Germany; 2 Faculty of Medicine, University of Freiburg, 79106 Freiburg, Germany; 3 Department of Public Health, School of Medicine, University of Naples Federico II, 80138 Naples, Italy; mpalumbomed@gmail.com (M.P.); sere.guerra@gmail.com (S.G.); alessandra_gallo@hotmail.it (A.G.); brunellazizolfi@hotmail.it (B.Z.); pgiampaolino@gmail.com (P.G.); giuseppe.bifulco@unina.it (G.B.); attiliodispiezio@libero.it (A.D.S.S.); m.chiaradeangelis88@gmail.com (M.C.D.A.)

**Keywords:** Uterine Tumor Resembling Ovarian Sex Cord Tumor (UTROSCT), hysteroscopy, hysterectomy, fertility-sparing, gene fusions, low malignant potential

## Abstract

Uterine Tumors Resembling Ovarian Sex Cord Tumors (UTROSCTs) are rare uterine mesenchymal neoplasms with uncertain biological potential. These tumors, which affect both premenopausal and postmenopausal women, usually have a benign clinical course. Nevertheless, local recurrences and distant metastases have been described. By analyzing 511 cases retrieved from individual reports and cases series, we provide here the most comprehensive overview of UTROSCT cases available in the literature, supplemented by two new cases of UTROSCTs. Case 1 was an asymptomatic 31-year-old woman who underwent a laparoscopic resection of a presumed leiomyoma. Case 2 was a 58-year-old postmenopausal woman with abnormal vaginal bleeding who underwent an outpatient hysteroscopic biopsy of a suspicious endometrial area. In both cases, immunohistochemical positivity for Calretinin and Inhibin was noted, typical for a sex cord differentiation. In both cases, total laparoscopic hysterectomy with bilateral salpingo-oophorectomy was performed. In light of the available literature, no pathognomonic clinical or imaging finding can be attributed to UTROSCT. Patients usually present with abnormal uterine bleeding or pelvic discomfort, but 20% of them are asymptomatic. In most cases, a simple hysterectomy appears to be the appropriate treatment, but for women who wish to become pregnant, uterus-preserving approaches should be discussed after excluding risk factors. Age, tumor size, lymphovascular space invasion, nuclear atypia, and cervical involvement are not reliable prognostic factors in UTROSCT. The current research suggests that aggressive cases (with extrauterine spread or recurrence) can be identified based on a distinct genetic and immunohistochemical phenotype. For instance, UTROSCTs characterized by GREB1::NCOA1-3 fusions and PD-L1 molecule expression appear to be predisposed to more aggressive behaviors and recurrence, with GREB1::NCOA2 being the most common gene fusion in recurrent tumors. Hence, redefining the criteria for UTROSCTs may allow a better selection of women suitable for fertility-sparing treatments or requiring more aggressive treatments in the future.

## 1. Introduction

Uterine Tumors Resembling Ovarian Sex Cord Tumors (UTROSCTs) are rare mesenchymal neoplasms uniquely classified within the group of “Mesenchymal tumors specific to the uterus” with “unspecified, borderline, or uncertain behavior” according to the WHO [1] and ICD-O [2] classifications. Their histologic features recapitulate the appearance of ovarian sex cord tumors [1]. The initial UTROSCT description dates back to 1945 when Morehead and Bowman reported a uterine neoplasm closely resembling a granulosa cell tumor in a 44-year-old woman [3]. A defining series published in 1976 by Clement and Scully detailed this novel histopathological entity [4]. Based on the quantity of sex cord elements, these tumors have been categorized into two subtypes: type 1 and type 2. Type 1 tumors, referred to as endometrial stromal tumors with sex cord-like elements (ESTSCLE), display a higher malignant potential compared to type 2 tumors. The latter encompasses classic UTROSCTs, which generally exhibit low-grade malignant potential with typically benign behavior, albeit with occasional recurrences. In 2008, Czernobilsky introduced an immunohistochemical algorithm that established the diagnostic criteria for UTROSCTs [5]. Four antibodies, including Calretinin, Inhibin, CD99, and Melan A, have been recognized as the most characteristic markers for confirming a UTROSCT diagnosis (type 2 tumors), while ESTSCLE typically only expresses a single sex cord marker, predominantly Calretinin [5]. Some authors, noting morphological and molecular distinctions, as well as differing clinical behaviors, argue against conflating ESTSCLE and UTROSCT into a single tumor category [6], leading to some studies excluding ESTSCLE, while others including it. Recent molecular discoveries support considering UTROSCT as a separate entity; however, the cellular precursor of UTROSCT remains unknown [7]. 

To date, UTROSCT is among the rarest uterine tumor types [1,4,5,7,8], with no literature review covering more than 80 cases. The established knowledge about UTROSCT is summarized in Table 1.

The diagnosis and treatment of UTROSCTs present challenges due to their rarity and the fact that their symptoms and imaging findings closely mimic those of leiomyomas. The impossibility of conducting large prospective studies and the limited feasibility of long-term follow-up observations often lead to intuitive treatment protocols, which can potentially result in overtreatment. Additionally, there is a low (20–25%) but significant risk of malignant progression in an ill-defined subset of UTROSCT cases. Therefore, there is an urgent need to refine histological criteria and prognostic factors by incorporating recent molecular findings. This will enable personalized treatment protocols that avoid overtreatment and reduce patient uncertainty, especially in cases with benign clinical behavior, and will clearly delineate tumors with aggressive potential.

In this review, by analyzing 511 cases from individual reports and case series, we offer the most exhaustive overview of UTROSCT to date. Based on a data set spanning 78 years, including significant advancements from 2022–2023, we emphasize the necessity of updating diagnostic and prognostic criteria to incorporate molecular tumor characteristics. Moreover, we propose new criteria for fertility-sparing treatments, marking a significant contribution in this field. In addition, we report two cases of UTROSCTs, one identified through hysteroscopic biopsy and another during laparoscopic surgery, both of which were managed with laparoscopic hysterectomy with bilateral salpingo-oopherectomy (BSO). 

## 2. Case Reports

### 2.1. Case 1 

**Figure 1:**
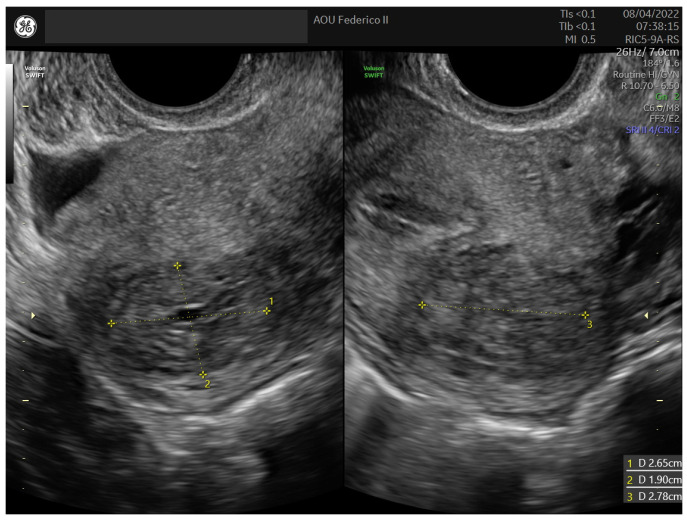
Sonographic appearance and measurements in three dimensions of the UTROSCT in Case 1.

**Figure 2:**
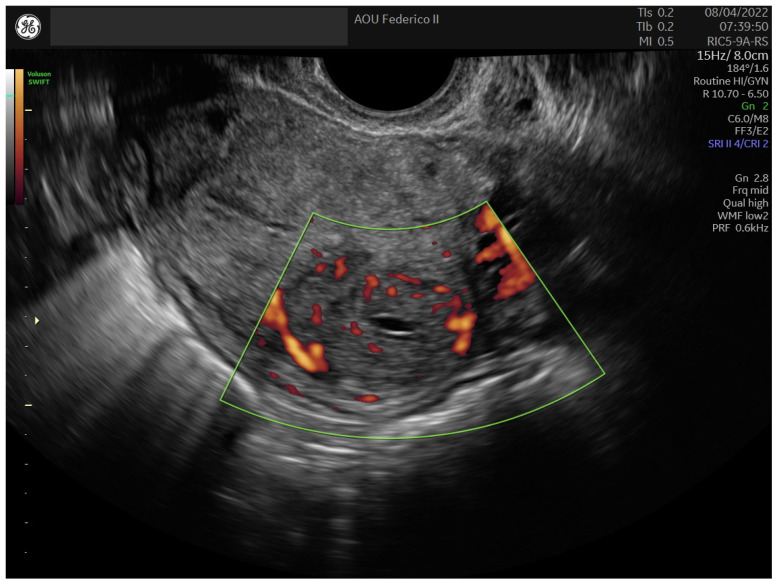
Sonographic Power-Doppler appearance of the UTROSCT in Case 1.

In 2022, a 31-year-old asymptomatic woman was referred to our center (University of Naples Federico II) for a routine gynecological ultrasound check. The patient had two spontaneous deliveries and experienced two spontaneous abortions; therefore, she had no desire for further pregnancies. She reported regular menstrual cycles in terms of duration and quantity and had no family history of cancer. At the time of admission, the patient had a BMI of 23.8 kg/m^2^. Transvaginal and transabdominal ultrasound examination, performed by an expert ultrasound gynecologist (B.Z.), revealed a normal anteverted uterus with regular margins and an inhomogeneous echo pattern. The endometrial echo pattern was regular with a normal endometrial thickness. Both ovaries appeared regular in size, shape, and echotexture. A well-defined, oval-shaped mass measuring 27.8 × 26.5 × 19 mm occupied the posterior uterine wall. The mass showed regular margins and non-uniform echogenicity due to the presence of some anechoic cystic areas (Figure 1). Moderate edge shadowing was present, while fan-shaped shadowing was absent. On color Doppler ultrasound, the lesion appeared not to be richly vascularized. No pelvic fluid was observed (Figure 2). A diagnostic hysteroscopy performed by the expert hysteroscopist (A.D.S.S.) revealed a regular uterine cavity without myoma imprints.

**Figure 3:**
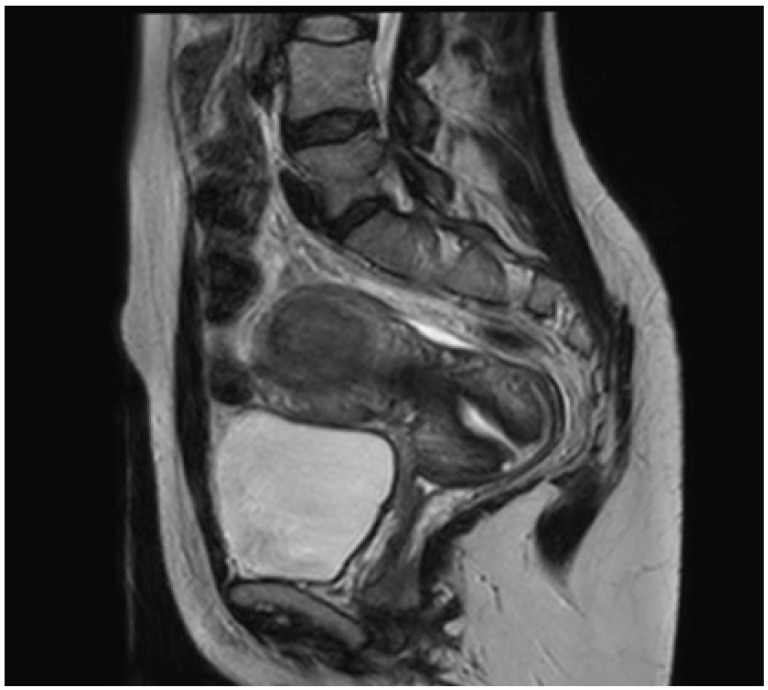
Magnetic resonance imaging of the UTROSCT in Case 1.

The radiological examination was performed externally by the patient and then was examined by an expert gynecologist oncologist (G.B.). Pelvic MRI confirmed the presence of an intramural-subserous “myoma” measuring 34 × 30 mm, with a screening intermediate signal T1-T2 and a peripheral ring of low signal T2. The mass was hypovascularized and exhibited a contextual millimeter cystic areola (see Figure 3). A red degeneration of uterine leiomyoma was suspected. 

**Figure 4:**
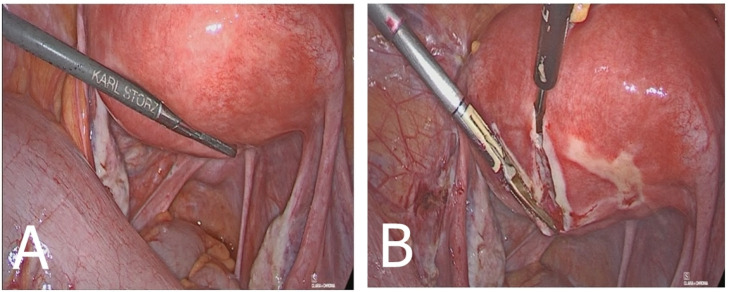
Intraoperative view: (**A**) typical intramural leiomyoma with a slight serosal protrusion of the posterior uterine wall and smooth Douglas peritoneum and (**B**) after incision, the tumor still presents as an intramural myoma.

Consequently, a laparoscopic myomectomy was conducted by an experienced gynecological surgeon (P.G.). The exploration of the abdomen and of the pelvis showed no macroscopic abnormalities; however, the uterus appeared irregular in shape for the presence of the uterine posterior mass of about 30 mm (Figure 4). The surgical specimen was extracted using an endobag to prevent dispersion in the abdominal cavity. 

Surprisingly, the mass was diagnosed as an UTROSCT upon the final pathological examination. Microscopic examination revealed a hypercellular tumor with a solid growth pattern and focal glandular and trabecular differentiation. The cells were small to medium in size, with scant cytoplasm and regular, ovoid nuclei. Mitotic activity was low, with approximately 2 mitoses per 10 high-power fields, and there was no evidence of necrosis. An infiltrative growth pattern into the myometrium was apparent, and this was suggestive of lymphovascular space invasion.

As described in Table 2, the immunohistochemical examination demonstrated tumor cells positivity for ER, PR, WT1, Calretinin, CD56, CD99, Smooth Muscle Actin, and Desmin, with focal positivity for E-cadherin and p16. The tumor was negative for Cyclin D1, BCOR, EMA, CK7, TTF1, GATA3, Chromogranin, Synaptophysin, Caldesmon, Cathepsin k, and Inhibin. Following the immunohistochemical criteria outlined by Czernobilsky [5], the co-expression of Calretinin and at least one other sex cord marker confirmed the diagnosis of UTROSCT (type 2).

Despite the patient’s young age and her lack of desire for fertility, a total laparoscopic HE with BSO was planned after multidisciplinary consultation.

### 2.2. Case 2 

**Figure 5:**
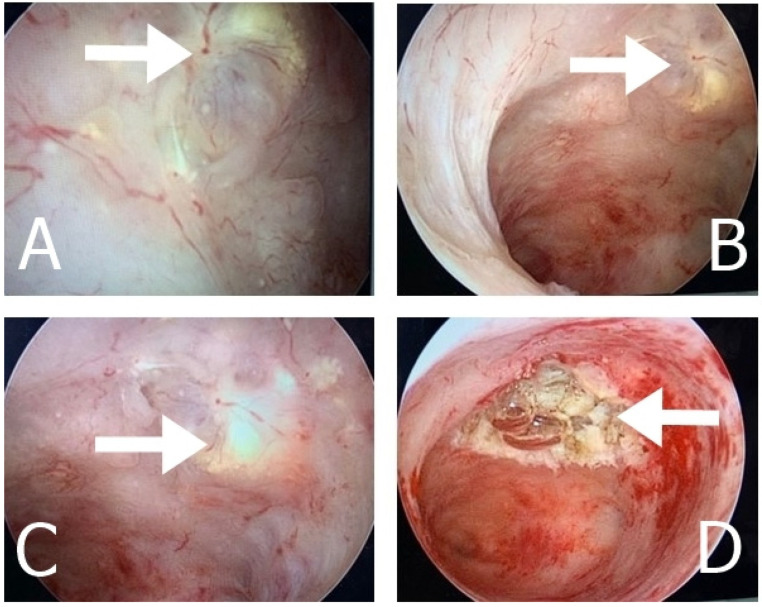
The arrows indicate the intraoperative appearance of UTROSCT on the anterior uterine wall (**A**–**C**) during hysteroscopy, and the post-biopsy view (**D**) of Case 2.

In 2019, a 58-year-old post-menopausal multiparous woman presented with abnormal vaginal bleeding that did not respond to medical therapy. Sonographic examination revealed an increased uterine volume, with two fibroids located in the anterior and posterior uterine wall, measuring 3.5 cm and 5 cm, respectively, and an endometrial thickness of 8 mm (despite the patient’s postmenopausal status). The woman underwent an ambulatory hysteroscopy (Naples), utilizing a vaginoscopic approach with a 5 mm continuous-flow hysteroscope (Bettocchi Office Hysteroscopes; KARL STORZ, Tuttlingen, Germany) equipped with an incorporating 5-Fr operating channel. During the procedure, an area of suspicion was identified on the anterior wall of the endometrium, measuring 1.5 cm, characterized by an irregular, yellow-colored surface. The presence of diffuse hypervascularization along with areas of necrosis was suggestive of malignancy, and a grasp biopsy was performed (see Figure 5).

The microscopic examination of the tissue revealed stretched and branched endometrial glands, surrounded by cells with abundant and foamy cytoplasm, suggesting the presence of sex cord-like elements. Following the recommendations of the pathology team, a second hysteroscopic examination was conducted to obtain deeper tissue samples. Using a 5-Fr Twizzle Versapoint™ bipolar system (Olympus, Hamburg, Germany) inserted through the operating channel of the hysteroscope, the suspicious area was completely resected (Figure 5D). The subsequent immunohistochemical studies were performed, confirming that the tumor was diffusely positive for Calretinin and Inhibin, focally positive for CD99, and negative for CD10 (see Table 2). Based on the immunohistochemical phenotype suggestive of the presence of sex cord components, the tumor was diagnosed as a UTROSCT (type 2). Staging examinations revealed neither locoregional spread nor distant metastases. Finally, a laparoscopic total HE with BSO was performed by the surgical-gynecological team (G.B. and P.G.), and the final histological diagnosis confirmed UTROSCT. The post-operative course was uneventful, and the patient was discharged on the third post-operative day, with a total regression of the initial symptoms.

Currently, three years after the initial diagnosis, the patient is relapse-free.

## 3. Literature Review

### 3.1. Materials and Methods

This exhaustive review meets the criteria of a scoping review as outlined by Paré et al.: (a) a broad scope of questions, (b) a comprehensive search strategy, (c) the inclusion of both conceptual and empirical primary sources, (d) explicit study selection criteria, (e) the absence of quality appraisal, and (f) the lack of meta-analytic tools, distinguishing our review from both narrative and systematic reviews [9]. Its cornerstone is the literature collection compiled by the first author (R.W.) over the last ten years. Consequently, the review was not prospectively registered per protocol. The final literature selection occurred during several rounds of literature searches performed in 2023: an electronic search of databases PubMed, SciELO, and Scopus; the scientific search engine Google Scholar; and publisher platforms such as ScienceDirect, Wiley Online Library, Taylor & Francis Online, Nature Publishing Group, SAGE Publications, and SpringerLink, was conducted up to November 2023. This was complemented by a meticulous review of reference lists. Our search algorithm combined terms like “uterine tumor resembling ovarian sex cord tumors”, “UTROSCT”, “ESTSCLE”, “sex-cord”, and “sex-cord like” with all relevant counterparts such as “immunohistochemistry”, “diagnosis”, “fertility-sparing”, “treatment”, “myomectomy”, “hysteroscopy”, “laparoscopy”, “ultrasound”, “imaging”, etc. There were no restrictions on language or geographic location. We recorded relevant aspects of each article, with special emphasis on histopathological findings, types of treatment applied, and reported outcomes. Our exclusion criteria included cases with no clinical information for data extraction, such as those lacking details on symptoms and/or survival, as well as duplicate reports. However, conference abstracts providing relevant information (in at least three categories) and published as supplements to established scientific journals were included. Two cases reported as UTROSCTs were excluded due to an immunohistochemical profile and a histological appearance that were not compatible with those of UTROSCT. Age differences between study groups were analyzed using the Student’s *t*-test, with a two-sided *p*-value of ≤ 0.05 considered statistically significant. Statistical analyses were conducted using JASP statistical software v.0.17.3 for Windows. 

### 3.2. Characteristics of Included Publications

We selected 104 case reports (studies with up to 3 cases) [3,10,11,12,13,14,15,16,17,18,19,20,21,22,23,24,25,26,27,28,29,30,31,32,33,34,35,36,37,38,39,40,41,42,43,44,45,46,47,48,49,50,51,52,53,54,55,56,57,58,59,60,61,62,63,64,65,66,67,68,69,70,71,72,73,74,75,76,77,78,79,80,81,82,83,84,85,86,87,88,89,90,91,92,93,94,95,96,97,98,99,100,101,102,103,104,105,106,107,108,109,110,111,112] and 24 case series (including between 4 and 75 cases) [4,6,113,114,115,116,117,118,119,120,121,122,123,124,125,126,127,128,129,130,131,132,133,134]. For a better readability, the cases are summarized in Table 2 (studies reporting 1–3 cases without recurrence), Table 3 (individual aggressive cases with extrauterine spread or recurrence), Table 4 (all series with more than 4 cases), and Table A1 (detailed data on patients who became pregnant with or after UTROSCT). The publications were mostly in English, two in German [15,79], two in Portuguese [23,48], and one in Spanish [19].

What makes our review the largest available review on UTROSCT is that we identified a total of 511 UTROSCT cases, including 93 individual cases with benign behavior (no extrauterine growth at the first diagnosis or recurrence, or with no reported recurrence) as listed in Table 3, 28 individual cases with aggressive behavior (extrauterine spread or metastasis at first diagnosis, or recurrent disease) listed in Table 4, and a further 373 cases reported in case series (starting with the seminal study by Clement and Scully [4] with 14 cases, up to the largest cohorts by Boyraz et al. [129] with 75 cases and Moore and McCluggage [122] with 34 cases), as shown in Table 5.

The year 2023 could be groundbreaking for UTROSCT research, as until November 2023, six series with a total of 156 cases, focusing on novel genetic and immunohistochemical insights, as well as 7 reports (including the present study) with a total of 8 cases, were published.

Some studies did not differentiate between UTROSCT type 1 and type 2, others included only type 2, and some studies used criteria for sex cord elements different from those of Czernobilsky [5]. In addition, recent research indicates that, in light of genetic heterogeneity, the dichotomous classification may be obsolete. With these facts in mind, we included both types of UTROSCTs to maintain comparability between older and newer studies.

### 3.3. Patient Characteristics 

**Figure 6:**
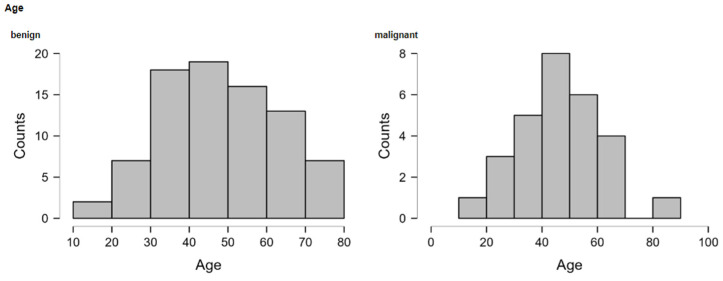
Age distribution of benign and clinically aggressive cases.

For patients reported individually, the mean age of women with a non-aggressive disease course (48.7, SD 14.66, range 18–77 years) and malignant disease course (46.8, SD 15.4, range 18–68 years) did not differ significantly (*p* = 0.53). Women aged 40 or younger accounted for 33% (31/93) of benign cases and 32% (9/28) of clinically aggressive cases. The age distribution is displayed in Figure 6.

The mean age and age range in our evaluation are similar to those reported in the landmark 1976 study by Clement and Scully, as well as in recent case series, e.g., Boyraz et al., 53 years (range 21–84) [129], and Goebel et al., 49.6 years (range 20–74) [125]. The youngest patient (12 years old) and the oldest (86 years old) were reported in the large case series by Moore and McCluggage [122].

The proportion of benign to malignant disease courses in our evaluation was 3:1, acknowledging the possibility of publication bias. There were 19 nulliparous women in the cohort, presenting a therapeutic challenge due to the uncertainty about the safety of fertility-sparing treatments and the dilemma of the potential overtreatment, including unnecessary hysterectomy and oophorectomy [114,127].

### 3.4. Clinical Presentation

The most common symptom across all ages and menopausal statuses was abnormal uterine bleeding (AUB), followed by pelvic pain or abdominal discomfort. In some cases, the tumor was asymptomatic and discovered incidentally during routine check-ups or infertility evaluations [25,50,56]. Hormonal disturbances such as galactorrhea [95], hyperprolactinemia [95,107], or hypercalcemia [20], resulting from ectopic prolactin or PTH-related peptide production, were the initial symptoms in other cases. Notably, in two cases, an emergency involving intraabdominal bleeding from the tumor led to the diagnosis of UTROSCT [89,93].

UTROSCTs typically mimic leiomyomas in their submucosal or intramural presentation. In around 15% of cases, the tumors present as intracavitary polyps. Pretherapeutic curettage sometimes yielded falsely negative results [75,76,111] or abnormal but misleading findings, such as low-grade endometrial stromal sarcoma (LG-ESS) [49,92,110], rhabdoid tumor [52], carcinosarcoma [14], or adenocarcinoma [100]. The value of preoperative targeted biopsy remains uncertain. It can either provide the definitive result (as in our Case 2 or [56]) or be misleading, as in [100], where image-guided omental biopsy revealed high-grade adenocarcinoma suggestive of epithelial ovarian carcinoma. The reason for the uncertainty may be the heterogenous composition of the tumor itself or a sampling error due to the presence of several similar lesions. 

In all but one reported cases, the diagnosis of UTROSCT was always unexpected and was made through the evaluation of the final surgical specimen. Intriguingly, the intraoperative appearance (via hysteroscopy or laparoscopy) often did not alter the initial assumption of the lesion being a leiomyoma or a polyp. 

In only one case was UTROSCT suspected preoperatively, based on a cervical liquid biopsy. However, even in that case, the diagnosis was facilitated by the presence of a polypoid tumor protruding into the vagina, and it was confirmed using extensive immunohistochemical staining, as the initial diagnosis was “atypical glandular cells consistent with adenocarcinoma, NOS” [69]. 

The coexistence of UTROSCT with other tumors at the time of surgery was not unusual, mostly with typical leiomyomas [18,27,41,42,43,45,48,60,103], but also with other neoplasms such as a second UTROSCT [29,60], ovarian sex cord stromal tumors [41], gastrointestinal stromal tumors [94], endometrial adenoacanthoma [17], cervical intraepithelial neoplasia [120], or cervical metastasis from breast cancer [40]. 

Information on the diagnostic utility of tumor markers for UTROSCT is scarce. Elevated CA-125 (up to 2210 U/L in [100]) levels have been occasionally reported [60,88,100,135], linking with extrauterine tumor spread [60,100,110] or accompanying conditions like adenomyosis [135]. Notably, CA-125, HE4, and CEA, which were normal in some reports [66,77,111], are not typical markers for sex cord tumors. Interestingly, serum Inhibin levels, a marker for sex cord tumors, have not been reported, reflecting the oversight of sex cord differentiation during perioperative consideration. Nevertheless, if elevated at the initial diagnosis, tumor markers (CA125, prolactin) might be useful for monitoring recurrence and response to therapy [100,110].

The usefulness of imaging in UTROSCT is limited, presenting no specific sonomorphologic or MRI features that would facilitate preoperative diagnosis. While there are detailed MRI reports, none have resulted in the correct preoperative identification of UTROSCT [17,30,49,71,72,81]. Intratumoral cystic degeneration, intratumoral hemorrhage, and necrosis are often seen on MRI, but are not pathognomonic [81], and can be mistaken for a liquid degeneration within a leiomyoma [63,71]. Unfortunately, imaging has sometimes led to the incorrect suspicions of UTROSCT relapse. For example, Hermsen et al. [49] reported a suspected early myometrial recurrence during pregnancy, which was monitored using MRI until the 34th week of gestation. The patient underwent a cesarean hysterectomy due to the presumed recurrence, but the lesion turned out to be adenomyosis. Carbone et al. [127] described lymphadenectomies performed due to suspected lymph node metastasis on imaging, which were not confirmed histologically.

Few cases were diagnosed with distant metastases at the time of diagnosis (see Table 4). Intraabdominal relapse and pulmonary metastases were the most common relapse sites [4,93,101,114,122]. Among all 511 cases, 18 patients died from or with the disease [4,93,100,111,112,114,122,129,130]. Those who died often experienced rapid disease progression, were metastatic at diagnosis, or progressed despite adjuvant therapies [93,111,112]. This raises the question of whether the application of unproven chemotherapeutic or hormonal therapies in UTROSCT could potentially worsen prognosis. Generally, survival data should be interpreted with caution due to the uncertain degree of underreporting, as follow-up times, when available, were often reported in months rather than years.

### 3.5. Gross and Ultrastructural Appearances

The mean size of tumors behaving benignly was 5.4 cm (SD: 4 cm, range: 0.9–20 cm), and for those behaving aggressively, it was 6.6 cm (SD: 3 cm, range: 1.5–11 cm), which was not significantly different (*p* = 0.27). Tumor growth limited to the cervix was observed infrequently, in approximately 10 cases. Most tumors presented as yellow to tan-yellow, tan-pink, or tan-gray masses, occasionally with hemorrhage or cystic areas. Microscopically, most tumors were well-circumscribed, but up to one-quarter exhibited an infiltrative growth pattern [121,129]. By definition, UTROSCTs mainly comprise cells resembling ovarian sex cord elements which are arranged in cords or trabeculae, or form tubular structures with central lumina. Occasional cases demonstrate retiform appearances [116]. Indeed, in the largest series by Boyraz et al., the following architectural patterns were present: cords, diffuse, hollow tubules, nests, trabeculae, retiform, solid tubules, pseudoangiomatoid, pseudopapillary, and whorled [129]. Typically, more than one pattern was seen. Cytologic atypia ranged from absent to mild in the majority of cases, and it was moderate in 21% and moderate-to-severe in 2.7% of tumors [129].

UTROSCTs exhibit a diverse immunohistochemical profile reflecting their complex histogenesis. The widely accepted immunohistochemical signature of UTROSCT is defined by a panel comprising Calretinin, Inhibin, CD99, and Melan A—markers indicative of sex cord lineage. Positivity for Calretinin, in conjunction with at least one other marker from this panel, is diagnostic for UTROSCT, whereas ESTSCLE generally express a single sex cord marker, predominantly Calretinin [5]. Other popular antibodies used in the diagnosis of UTROSCT are those immunoreactive for mesenchymal and epithelial elements, including Vimentin, Desmin, Cytokeratin, Epithelial Membrane Antigen (EMA), CD10, and estrogen/progesterone receptors (ER/PR) [5,41]. However, several studies rely on alternative algorithms [42,96,97,100,101,135,136], considering tumors such as UTROSCT to be negative for Calretinin but positive for other sex cord markers. Nogales et al. emphasize that UTROSCTs are not “a discrete entity but a group of tumors that do not necessarily have a stereotyped morphology and are only defined by comparison with histologically equivalent ovarian tumors. Morphologically, they imperfectly reproduce the histology and immunohistochemistry of a variety of patterns (trabecular, tubular, pseudoglandular, luteinized, etc.) of sex-cord stromal tumors of the ovary. Moreover, some exhibit a mixed sex cord and predominantly myoid phenotype, which can also be part of the tumor proliferation in sex-cord-like structures of UTROSCTs” [137]. 

Some authors favor CD56 as the most reliable immunohistochemical UTROSCT marker [41,42]. A review on the immunohistochemical features of the 44 cases of UTROSCT reported by Abdullazade et al. showed CD56 expression in 100%, followed by positivity for Calretinin in 94%, AE1/AE3 in 73%, CD10 in 50%, Inhibin in 49%, Desmin in 46%, EMA in 29%, and Caldesmon in 7% of cases [41]. In addition, Stewart et al. explored both older and newer immunohistochemical markers of sex cord-like elements in UTROSCT and confirmed that Calretinin was more sensitive than Inhibin, FOXL2, and steroidogenic factor-1 (SF1), but SF1 was the only marker specific to UTROSCT, as it was negative in all potential histological mimics that were investigated [121]. These results were confirmed by Croce et al., who found that 53% (10/19) of investigated UTROSCT samples exhibited nuclear immunoreactivity with FOXL2, and 58% (11/19) showed nuclear staining with SF1 [123].

Apart from the polyphenotypic histomorphologic appearance of UTROSCT itself, in some cases, UTROSCT displayed partial sarcomatous features [77], myxoid features [87], or osteoid metaplasia [12]. Conversely, UTROSCT elements can be incorporated into other tumors, e.g., adenomyosis [135], true endometrial polyps [138], endometrioid carcinomas [139], or LG-ESS [136,140]. Two intriguing cases reported 32 years apart (1989 and 2021) involved the initial tumors diagnosed (or misdiagnosed?) as LG-ESS, but recurrences with significantly abundant sex cord-like elements met the diagnostic criteria of UTROSCT [92,110]. In the first case, speculation about the misdiagnosis of the initial tumor could be justified (even the report’s title uses of confusing terminology); however, the latter case is substantiated by meticulous molecular analysis showing a UTROSCT-typical genetic rearrangement in the recurrent tumor (GREB1-NCOA2 fusion) [110].

### 3.6. Gene Fusions in UTROSCT 

Recent studies have highlighted the significant role of specific gene fusions in UTROSCT, particularly those combining genes relevant to sex hormone pathways with (co)activator oncogenes. These include estrogen receptor 1 (ESR1) and growth regulation by estrogen in breast cancer 1 (GREB1) genes, which fuse with nuclear receptor coactivators NCOA1-3 [76,104,105,124,125,131,134]. GREB1 encodes for a protein driven transcriptionally by estrogen-bound ER, being a crucial component of the canonical estrogen/ER signaling pathway. Binding with estrogen, ESR1 is essential for a broad range of physiological functions, but is also involved in pathologic processes, including breast cancer, endometrial cancer, or osteoporosis [76,104,105,132,133,134]. Mutations in ESR1′s ligand-binding domain have been correlated with resistance to hormone therapy in ER-positive breast cancer. Notably, UTROSCTs with ESR1 rearrangements may be resistant to estrogen blockade as the ER ligand-binding domain is lost in these fusions, potentially explaining the resistance to anti-hormonal treatments reported in relapsed UTROSCT [96,99,112].

The NCOA family, a part of the p160 steroid receptor coactivators (SRC1/2/3), interacts with ligand-dependent hormone nuclear receptors, including estrogen receptor-alpha (ERα). It mediates transcriptional programs promoting cellular proliferation, metabolism, growth, and survival [109]. In UTROSCTs with NCOA rearrangements, the chimeric fusion protein expressed is under the transcriptional control of the 5′ fusion partner promoter, retaining the 3′ NCOA fusion partner’s nuclear receptor co-activator and transcriptional activation domains [105,109,141]. Recent research has specifically focused on NCOA1, NCOA2, and NCOA3 gene fusions in UTROSCT [76,104,105,124,125,131,134]. Bi et al. reported that in recurrent UTROSCT cases, the GREB1::NCOA2 fusion was the most common, accounting for 57% of cases, with GREB1::NCOA1 and ESR1 fusions also detected. These GREB1-rearranged tumors were typically more advanced, larger, and occurred in older patients [132]. Lu et al. reported recurrent NCOA1-3 rearrangements in 87.5% (14/16) of their series, without JAZF1, PHF1, BCOR, or YWHAE rearrangements, underscoring the diagnostic value of these rearrangements in distinguishing UTROSCT from endometrial stromal tumors [131]. In Goebel et al.’s study of 26 UTROSCT cases, NCOA1/3 rearrangement was identified in 81.8% (18/22) of cases, with ESR1-NCOA3 being the most common fusion, followed by GREB1-NCOA1, ESR1-NCOA2, and GREB1-NCOA2 rearrangements. Only one case experienced recurrence 66 months after the initial diagnosis, and this was the only case with a GREB1-NCOA2 fusion [125]. In the recent case series by Quji et al., six types of fusion genes were identified: ESR1::NCOA3 (found in 4 cases), ESR1::NCOA2 (2 cases), ESR1::CITED2 (2 cases), GREB1::NCOA2 (2 cases), GREB1::NCOA1 (1 case), and GREB1::NCOA3 (1 case). Notably, the three cases with recurrence and metastasis were associated with the fusion genes GREB1::NCOA2, ESR1::NCOA3, and ESR1::CITED2 [134]. Additionally, Croce et al. reported a novel translocation t(2;3) involving GREB1 and CTNNB1 (encoding β-catenin), activating the Wnt/β-catenin signaling pathway and presenting a potential new therapeutic target [105,123]. These gene fusions, especially those involving NCOA genes, interact with hormone nuclear receptors and mediate essential cellular functions, hinting at the oncogenic potential when these are dysregulated [109]. The partner genes of GREB1- or ESR1-rearranged UTROSCT, including NCOA1–3, NR4A3, GTF2A1, and CTNNB1, are described in Table 6.

To mention the negative findings in UTROSCT, which can be useful in differentiation from other uterine neoplasms, it has been consequently shown that UTROSCTs lack the JAZF1-JJAZ1 translocation that is frequently seen in endometrial stromal tumors [87,97,117]. Furthermore, the Bcl-2 and MALT1 genes are unlikely to be involved in the pathogenesis of UTRSCT, although they are located close to the frequently observed translocation points t(X;6)(p22.3;q23.1) and t(4;18)(q21.1;q21.3) [22,31]. Finally, UTRSCTs frequently exhibit positivity for sex cord markers FOXL2 and SF-1 without showing any mutations in the FOXL2 and DICER1 genes [123].

### 3.7. Risk Factors and Prognostic Factors

Considering the small number of cases, there are no established risk factors for UTROSCT, and reports on their hereditary background are absent. Seven UTROSCT cases have been reported in patients treated with tamoxifen [28,36,40,46,70,116,126]. A causal association should be approached with caution, as the majority of UTROSCT cases developed without tamoxifen exposure, and the number of women exposed to tamoxifen—due to the high incidence of breast cancer—is disproportionately large in comparison to the rarity of UTROSCT. Furthermore, some gene rearrangements seen in UTROSCT (GREB1-fusions) are known to make the tumors responsive to tamoxifen therapy. 

Factors such as age, menopausal status, or the extent of surgery (whether uterus-preserving or not) have not been predictive of relapse. Accordingly, in our review, age and tumor size have not been associated with more aggressive disease course. 

Traditionally, based on the series by Clement and Scully, recurrences are more commonly associated with type 1 tumors than with type 2 tumors [4,113]. Certain histological features have been identified as prognostic factors in UTROSCT. Boyraz et al. observed five recurrences among 58 patients over an average follow-up time of 73 months (ranging from 22 to 192 months) and concluded that malignant UTROSCTs exhibited more than three of the following five features compared to their benign counterparts: size greater than 5 cm, at least moderate cytologic atypia, three or more mitoses per 10 high-power fields (HPF), infiltrative borders, and necrosis. One of the five malignant tumors displayed extensive rhabdoid morphology [129]. Additionally, tumor size, lymphovascular space involvement, nuclear atypia, cervical involvement, or the proliferation index (Ki67) could not be confirmed as prognostic factors in UTROSCT by various authors [54,91,94,95,97,98,99,122].

Myometrial invasion and serosal involvement are traditional pathological risk factors evaluated, though their utility in UTROSCT has been supported by individual observations without comparators [54,95,97,98,99]. In contrast, tumors with infiltrating growth patterns and no recurrences during follow-ups are well-documented [47,50]. Some immunophenotypes, like those with a predominant epithelial retiform component (RUTROSCT), seem to be associated with a good prognosis and could help in preventing overtreatment in selected patients [116]. Due to the relative overrepresentation of malignant cases in the dataset of Moore and McCluggae, their findings are particularly insightful regarding the malignant potential of individual UTROSCT cases, i.e., 8 of 34 patients (23.5%) developed extraterine metastases in various sites, including the pelvic and abdominal peritoneum, ovary, lymph nodes, bone, liver, and lung, and three patients (8.8%) died due to the tumors [122]. Neoplasms exhibiting malignant behavior that occurred on average in older patients were larger and more likely to show necrosis, lymphovascular invasion, cervical involvement, significant nuclear atypia, and significant mitotic activity. Finally, only the presence of necrosis and a significant mitotic activity were statistically significant [122]. 

Given the overlap in pathological parameters between clinically benign and malignant neoplasms, some authors proposed considering all UTROSCTs as potentially malignant until proven otherwise [122]. This somewhat fatalistic view has been significantly clarified in recent years by numerous studies equivocally pointing to specific genetic changes within UTROSCT as the most important predictors of malignancy and recurrence. Gene rearrangements involving key genes in sex hormone pathways appear to be the best predictors of recurrence. As demonstrated in [105,125], UTROSCTs with GREB1 rearrangement may have a high risk of recurrence or metastasis. Regarding prognosis, GREB1-rearranged tumors tended to occur in significantly older women than UTROSCT with ESR1 fusions; moreover, GREB1-rearranged tumors tended to be larger and more mitotically active and behave more aggressively [105]. Recently, Yin et al. [86] described novel fusion genes involving ESR1 and GREB1 as the 5′ partner and NCOA1-3 as the 3′ partner. Genotype and phenotype correlation has suggested that GREB1-rearranged UTROSCTs may have a higher tendency to behave aggressively.

Particularly, tumors with GREB1::NCOA2 fusions are more likely to recur than those with any other genetic alteration [132]. The suggestive study by Xiong et al. combined classical histomorphological, immunohistochemical, and molecular–genetic predictors, and finding a significant mitotic activity, a high expression of stromal PD-L1, and an NCOA2 gene alteration may help in identifying the subset of UTROSCT with aggressive behavior and shorter disease-free survival (DFS) [130].

### 3.8. Treatment Strategies

Total abdominal hysterectomy with BSO was the most common treatment, followed by total abdominal hysterectomy without BSO. Other forms of hysterectomy, such as (laparoscopically assisted) vaginal hysterectomy, were also reported [4,63,118,120].

For women who have completed their reproductive plans, a total HE, whether abdominal or laparoscopic, and depending on menopausal status, with or without ovariectomy, appears to be an adequate treatment for tumors confined to the uterus.

Supracervical hysterectomy, although mentioned incidentally in reports by Bakula-Zalewska et al. [119] and Carbone et al. [127], does not appear to be appropriate for UTROSCT because of its potential for cervical involvement and the need for tumor morcellation, which can increase the risk of recurrence [109]. The scarcity of intraoperative details in most cases and documented recurrences following tumor disintegration [58,60,109] should prompt surgeons to be particularly vigilant.

When bulky lymph nodes are present, removing the nodes and performing regional lymphadenectomy is a straightforward decision. However, for cases with histologically aggressive tumors in preoperative specimens, there is no established strategy. The use of indocyanine green for sentinel lymph node detection, while aligning with current trends to minimize perioperative morbidity [142], has not been specifically studied in UTROSCT. Consequently, routine systematic lymphadenectomy or non-specific sampling in UTROSCT lacks robust support from existing evidence. Individual decisions, taking into account patient preferences, are advisable in such scenarios.

Given the fact that around 15% of all reported cases occurred in nulligravidas, the possibility of uterus-preserving treatments and, on the other hand, the identification of tumors with aggressive behavior (where conservative treatments could potentially lead to worse prognosis) are critical. Confusingly, among nulliparous patients, only 30% underwent fertility-sparing treatments. Notably, the recurrence rate does not appear to be higher following fertility-sparing treatments compared to hysterectomy or more radical approaches. In the youngest reported case, a 12-year-old patient underwent uterus-sparing removal of a 19.5 cm tumor and remained recurrence-free at a 27-month follow-up [122]. Conversely, cases of nulliparous patients aged 18 [92] and 19 [114] who underwent hysterectomy and BSO raise concerns about the potential overtreatment.

### 3.9. UTROSCT and Fertility

Ten cases of pregnancy associated with UTROSCT have been reported, including two pregnancies in one patient both during and after UTROSCT treatment [127]. Interestingly, three cases of UTROSCT were identified during evaluations for primary or secondary infertility [25,50,56], and another case was detected following a miscarriage curettage [127]. In three instances, conception occurred while the tumor was present [93,127] or shortly after tumor resection [49]. Seven nulliparous women successfully conceived following fertility-sparing treatments for UTROSCT [25,34,49,50,56,58,127], and each experienced an uncomplicated pregnancy. Except for one in vitro fertilization [50], all pregnancies occurred spontaneously. One patient, diagnosed with an advanced tumor in the 35th week of pregnancy [93], died nine months later due to disease progression; however, all other patients remained alive. Some women underwent hysterectomy at or after delivery [49,58], while others did not [34,56,127]. These cases are summarized in Table A1.

A viable approach appears to be delayed hysterectomy, undertaken immediately after fulfilling reproductive plans. This strategy was employed in cases [49,50,58]. Schraag et al. reported a successful pregnancy in a patient who had undergone two organ-preserving treatments (initially for tumor persistence and subsequently for relapse), eventually followed by a hysterectomy [58]. Considering the potential for late local recurrences and the absence of long-term cohort studies, offering a hysterectomy upon the completion of family planning seems advisable [25,58].

### 3.10. Follow-Up

Follow-up protocols for UTROSCT differ between authors. Common imaging modalities like transvaginal ultrasound or MRI can be used, since the problem with UTROSCT is not that it is not visible on imaging, but only indistinguishable from common pathologies by ultrasound or MRI. Accordingly, most recurrences were detected through ultrasound or MRI. While there are no established serum tumor markers specific to UTROSCT, markers that were elevated at initial diagnosis and responsive to treatment should be monitored during follow-up. Notably, increases in serum CA125 [100,110] or prolactin [95,107] levels have preceded some recurrences.

In cases where the initial approach was hysteroscopic, performing repeat hysteroscopy to exclude intracavitary tumor residues after local resection [34] or as a part of follow-up can enhance the safety of conservative strategies [37,56]. Garuti et al. proposed a follow-up regimen of clinical examinations and transvaginal sonography at 6-month intervals, supplemented by office hysteroscopy every 12 months for the first three years [37]. whereas Similarly, De Franciscis et al. recommended transvaginal ultrasound examinations every six months and diagnostic hysteroscopy annually for five years [56]. The duration of follow-up remains undefined due to low number of recurrent cases, no established patterns of recurrence, and PFSs of 7 [112], 11 [96], 14 [110], 23 [99] or 32 [104] years being not uncommon. 

### 3.11. Recurrence Treatments

Surgery aimed at the complete removal of any tumor residues key to long-term survival, even in the cases of recurrence, with singular follow-ups reaching 32 years [104]. Cömert et al. [102] calculated the average recurrence rate of UTROSCT at 6.3%. The response to chemotherapy and hormonal treatments is generally poor. Reported chemotherapeutic regimens include ifosfamide, carboplatin, and CYVADIC (cyclophosphamide, vincristine, doxorubicin, and dacarbazine), with no response in a patient who subsequently died 9 months after the initial diagnosis [93]. BEP (bleomycin, etoposide, cisplatin) was used in a 43-year-old patient with recurrence [60], as well as megestrol acetate and letrozole, with recurrence occurring 3 months after the completion of adjuvant therapy. Letrozole and medroxyprogesterone acetate were abandoned after 3 months due to no response [99]. Blinmann et al. [96] applied tamoxifen, warfarin, and doxorubicin with minimal response and anastrazol with uncertain effect. Another patient received BEP and relapsed 8 months after the completion of therapy. They continued with carboplatin, paclitaxel, and ombrabulin, with repeated disease progression. The subsequent treatments included epirubicin with pazopanib, letrozole, trabectedin, paclitaxel, BI860585, and exemestane and rechallenged with epirubicin, BMS-986148, and nivolumab until the final progression and death [112].

## 4. Discussion

This review represents the largest aggregation of UTROSCT cases to date, with 511 cases reported from the initial description in 1945 to late 2023. We also contribute two new cases of UTROSCT: one mimicking a myoma FIGO type 4, diagnosed after laparoscopic “myomectomy,” and another manifesting as a submucous mass with endometrial thickening, diagnosed via office hysteroscopy with endometrial biopsy [143]. For both individuals, a hysterectomy was the chosen definitive treatment.

Although our review provides several insights into UTROSCT’s clinical presentation and management, the level of available evidence is still unsatisfactory, due to relying on case reports and retrospective case series (the largest comprising 75 cases). The available knowledge about UTROSCT suffers from insufficient case numbers and the impossibility of randomized controlled trials, but also publication bias. The latter shortcoming can lead to an overrepresentation of aggressive cases (as stated in [122]) or an assumption of cases to be prematurely benign, as cases with a follow-up of few months or simply not reporting a recurrence are commonly classified as “not recurrent” (the latter limitation may apply also to the present review). The quality of reporting, e.g., lacking histological description [71], further contributes to the uncertainty of available evidence.

AUB is the most common symptom for leiomyomas, endometrial polyps, and other endometrial proliferations [144]. The symptoms and the sonographic appearance of UTROSCT are identical with those of very common uterine pathologies responsible for AUB or pelvic pain. In addition, a significant proportion of UTROSCTs become asymptomatic, a characteristic shared with uterine myomas. The sonographic or MRI appearance is usually suggestive of uterine leiomyoma or, less commonly, adenomyosis. Neither the size (ranging from 1 to 20 cm), nor the relationship to the myometrium (whether submucosal or intramural) or endometrium (such as polypoid intracavitary growth), facilitates the differentiation of UTROSCT from myomas or polyps. Additionally, it is noteworthy that UTROSCT often coexists with one or more leiomyomas in the same patient [18,27,41,103]. 

The difficulty in differentiating between uterine tumors with a sonographic appearance similar to myomas is well known [145,146,147]. In the study by Russo et al., no significant differences were observed between benign and malignant lesions in terms of echogenicity, the presence of shadowing, or size; however, cystic areas within the lesion were seen in 31% of typical leiomyomas and in 55% of leiomyoma variants, adenomyomas, and smooth muscle tumors of uncertain malignant potential (STUMP) or leiomyosarcoma. Lesion borders were regular in 99% of benign lesions and 40% of malignant lesions [145]. Similarly, intratumoral cystic areas, poor or moderate vascularization, and the absence of shadowing were more common in sarcomas [146] and in STUMP [147]. In this context, the observation by Chiappa et al. that sparse edge shadowing and a lesser degree of vascularization, especially intralesional, might be more common in UTROSCT than in typical leiomyomas is not surprising. However, these findings cannot be interpreted as UTROSCT specific [73].

Our review confirmed that age and tumor size do not differ between patients with UTROSCT presenting aggressive behavior (defined by the initial extrauterine tumor spread or recurrence) and those with benign clinical course. The unspecific symptomatic and sonographic appearance, grossly overlapping with those of uterine myomas or intracavitary polyps, are the inherited features of UTROSCT and therefore not modifiable. Nevertheless, progress has been achieved in the areas of individualized therapy planning and immunohistochemical and molecular diagnosis. 

The analysis of gene fusions is a new tool in the differential diagnosis of UTROSCT, offering novel prognostic factors that facilitate tailored therapy planning. For example, UTROSCT differs from endometrial stromal neoplasms (including those with sex cord stromal differentiation) in that it typically does not exhibit the JAZF1-SUZ12 fusion or PHF1 rearrangements [7]. Furthermore, UTROSCT is not associated with FOLX2 and DICER1 mutations, which are indicative of ovarian adult-type granulosa cell tumors and Sertoli–Leydig cell tumors, respectively [7]. On the other hand, recent findings have identified recurrent fusions involving NCOA2 and NCOA3 (such as ESR1-NCOA3, ESR1-NCOA2, or GREB1-NCOA2) in UTROSCT. These genes, NCOA2 and NCOA3, are known to be involved in steroid hormone regulation, and the disruptions in their nuclear receptor coactivator domains are thought to play a crucial role in the development of UTROSCT [7,76,104,105,124,125,131,132,133,134].

Recent research has provided molecular features that aid in a more personalized approach. We assume that the future of UTROSCT diagnosis and treatment—including the decision to preserve the uterus or not—will be the molecular–genetic testing, as it has become common in other gynecologic malignancies, e.g., endometrial carcinoma [148,149]. This hypothesis is supported by numerous recent studies [86,105,109,123,124,125,126,128,129,130,131,132] that identified factors associated with aggressive clinical behavior and recurrence. In UTROSCT, GREB1 or ESR1 often fuse with members of the p160 steroid receptor coactivator family, which includes NCOA1, NCOA2, and NCOA3. These gene fusions, such as GREB1::NCOA2 and ESR1::NCOA2, result in the aberrant activation of estrogen signaling pathways, driving the proliferation and survival of tumor cells. The fusion proteins maintain the transcriptional activation function, which may lead to the dysregulated expression of genes that are normally regulated by sex hormones, potentially contributing to the tumorigenesis of UTROSCT. The specific fusion types, particularly GREB1::NCOA2, are associated with a higher recurrence risk. Furthermore, the GREB1 rearrangements can be detected both in aggressive primary and relapsed tumors [105,125]. Since specific gene fusions, a high expression of stromal PD-L1, and a significant mitotic activity have been shown to predict aggressive UTROSCT [130], we postulate to include these parameters into the standard evaluation of UTROSCT. Along with these findings, the most recent WHO classification concludes that “although data is limited, features that may be associated with aggressive behavior include a mitotic count >2 per 2 mm2 (>2 mitoses per 10 HPFs if field diameter is 0.55 mm), necrosis, extensive (>50%) rhabdoid morphology and potentially tumors with GREB1 rearrangement” [1].

### Criteria for Fertility-Sparing UTROSCT Treatments

A significant strength of the paper is the accumulation of cases with a favorable outcome despite uterus-preserving strategies. In 2015, Watrowski et al. recommended to consider uterus preservation—after counseling about the unpredictable course of the disease—in young patients with small, well-circumscribed tumors limited to the intrauterine cavity, with a hysteroscopic follow-up or at least a regular transvaginal ultrasound [47]. 

As of 2023, in light of the current literature, we suggest following criteria for considering fertility-preserving treatments:

- Desire for pregnancy.
- Evaluation and documentation of risk factors:
  ⚬ Tumor size/extrauterine spread;
  ⚬ Presence of necrosis;
  ⚬ Mitotic activity;
  ⚬ Presence of GREB1::NCOA-1/3 fusions.
- No tumor residues after last treatment (e.g., negative re-hysteroscopy).
- Possibility and adherence to follow-up.
- Offering hysterectomy after the completion of family planning.

## 5. Conclusions

Our review of literature comprises the largest data extraction from 511 cases, two of them being reported for the first time. UTROSCT is not associated with specific clinical presentations or pathognomonic findings; the symptoms and sonographic appearance of UTROSCT largely overlap with those of leiomyoma, and less commonly, with those of adenomyosis or endometrial polyps. Therefore, it is usually discovered accidentally, often after a disintegrating surgical modality performed for benign pathology. Fertility-preserving initial treatment does not seem to worsen the prognosis. Common parameters, like patient age, tumor size, lymphovascular space invasion, nuclear atypia, and cervical involvement, are not prognostic factors in UTROSCT. However, current research suggests that aggressive cases (with extrauterine spread or recurrence) can be identified based on a distinct genetic and immunohistochemical phenotype. Particularly, UTROSCT with GREB1::NCOA2 gene fusions or the expression of the PD-L1 molecule seem to be predisposed to metastasize and relapse. Hence, we advocate a subclassifcation of UTROSCT according to molecular criteria to allow a better selection of women suitable for fertility-sparing treatments and, on the other hand, with an increased risk of relapse, possibly requiring more radical treatments. 

## Figures and Tables

**Table 1:** Clinical and histological characteristics of UTROSCT (adopted from [7] and [8]).

Age Distribution	Adult women (third to sixth decades) Equal distribution among parous and non-parous women
Clinical Features	Abnormal uterine bleeding Pain Often asymptomatic
Gross Findings	Well circumscribed, variable size (median 6 cm; >10 cm in 20%) Infrequent cervical involvement (<10%) Infrequent extrauterine spread (<10%) Homogeneous yellow to tan cut surface
Microscopic Findings	Usually well-demarcated; irregular border with infiltration may occur Cords trabeculae, nests, gland-like structures, tubules, and retiform growth reminiscent of sex cord stromal tumors Bland, uniform cells with scant to abundant eosinophilic cytoplasm Relatively frequent: mitosis (56%), atypia (40%), and plemorphism (40%) Infrequent: lympho-vascular space invasion (<10%) and necrosis (<7%)
Immunohistochemical Features	Calretinin (>95%), CD99 (>90%), CD 56 (>90%), inhibin, WT1, and FOXL2 positive ER (>70%) and PR (90%) positive Smooth muscle markers, Melan-A, CD10, and epithelial markers frequently positive SF1 can be positive HMB45 negative
Differential Diagnosis	Endometrial stromal tumor (nodule or low-grade sarcoma) Endometrial carcinoma with sex cord differentiation Perivascular epithelioid cell tumor Epithelioid smooth muscle tumor

**Table 2:** Immunohistochemical characteristics of tumors.

Antibody	Marker for	Case 1 (31 y.o.)	Case 2 (58 y.o.)
Calretinin	Sex cord	Positive (++)	Positive (++)
CD99	Sex cord	Positive (++)	Focally positive (+)
CD56 (NCAM)	Sex cord	Positive (++)	N/a
Inhibin	Sex cord	Negative	Positive (++)
Wilms tumor protein (WT1)	Mesothelioma/serous differentiation	Positive (++)	N/a
Cytokeratin (CK 7)	Epithelium (e.g., ovarian adenocarcinoma)	Negative	Negative
EMA	Epithelial membrane	Negative	Negative
Alpha-SMC	Smooth muscle cells	Positive (++)	Focally positive (+)
Desmin	Muscle-type intermediate filaments	Focally positive (+)	Focally positive (+)
Caldesmon	Myogenic marker	Negative	Negative
E-Cadherin	Epithelial marker	Focally positive (+)	Focally positive (+)
P16	HPV-related carcinomas	Focally positive (+)	Negative
Cyclin D1	Different cancers	Negative	Negative
BCOR	Soft tissue sarcomas, ESS	Negative	Negative
TTF1	Lung adenocarcinoma, thyroid carcinoma	Negative	Negative
GATA3	Breast cancer	Negative	Negative
Chromogranin	Neuroendocrine marker	Negative	Negative
Synaptophysin	Neuroendocrine marker	Negative	Negative
Cathepsin K	Breast, lung, prostate, kidney	Negative	Negative
Estrogen receptor	Genital and breast carcinomas	Positive (++)	Positive (++)
Progesterone receptor	Genital and breast carcinomas	Positive (++)	Positive (++)

alpha-SMA: alpha-Smooth Muscle Actin, ER: estrogen receptor, PR: progesterone receptor, WT1: Wilms tumor 1, BCOR: BCL6 corepressor, EMA: epithelial membrane antigene, CK7: cytokeratin 7, TTF1: thyroid transcription factor-1. NCAM: neural cell adhesion molecule (=CD56); and ESS: endometrial stromal sarcoma; (+) and (++) indicate the intensity of staining, with ‘(+)’ denoting a lower intensity and ‘(++)’ indicating a higher intensity.

**Table 3:** Case reports with benign outcome (no evidence or no reporting of extrauterine spread or relapse).

First Author	Year	Age	Parity	Symptom	Site	Size, cm	Treatments	FU (mo)	Associated with
Morehead [3]	1945	44	G7, P5	AUB	Uterine mass	2	vaginal HE	N/a	
Tang [10]	1979	28	N/a	AUB	Intramural	9	HE	8	“indistinguishable from UTROSCT” (but referred to as “stromomyoma”)
Fekete [11]	1985		N/a		Submucosal		HE	N/a	
Iwasaki [12]	1986	33					TAH		Osteoid metaplasia
Erhan [13]	1992	40	N/a	pain	Intramural	9	TAH, BSO		Stromomyoma; D&C: normal
Moll [14]	1992	73	G2/P1	AUB, pain	Polypoid	5	TAH, BSO	12	D&C: carcinosarcoma
Horn [15]	1995	54	N/a		Uterine mass	7.6	HE, BSO	27	Partially retroperitoneal
Miliaras [16]	1997	57	N/a	AUB	Intramural	7.5	HE, BSO	30	
Okada [17]	2001				Intramural				Endometrial adenoacanthoma
Hauptmann [18]	2001	49	N/a	AUB	Intramural	4.5	HE	7	Multiple leiomyomas
Ribau Díez [19]	2001	36	G3, P3	AUB	Uterine mass	5.7	HE	94	
Suzuki [20]	2002	66	G1, P1	Hypercalcemia	Cervix	8	HE, BSO	N/a	Hypercalcemia, hyper-PTH-emia
Kuruvila [21]	2003	50	N/a	AUB	Polypoid		D&C in both cases	12	
Wang [22]	2003	34	G2, P1	AUB	Submucosal	4.7	HE, BSO	12	
Franco [23]	2003	69	G7, P4	AUB	Uterine mass	7	D&C, HE, BSO, LN sampling	N/a	
Kabbani [24]	2003	24	G0, P0	AUB	Cervix	11	HE, irradiation, PLN sampling	12	
Hillard [25]	2004	32	G0, P0	AUB	Intramural	N/a	Lsc TR	15	**Pregnancy** after treatment
Sutak [26]	2005	72	N/a	AUB	Intramural	2.2	HE + BSO	15	
Motiwala [27]	2006	63	N/a	AUB	Intramural	11	HE + BSO	N/a	Multiple leiomyomas
Oztekin [28]	2006	58	G13, P3	Pain	Intramural	6	TAH, BSO	8	Tamoxifen therapy
Zámecník [29]	2006	39	N/a	N/a	Intramural	2	HE	N/a	Double tumor
Calisir [30]	2007	65	N/a	Pelvic mass	Intramural	8.5	HE, BSO	N/a	Mazabraud’s syndrome
Sitic [31]	2007	76	N/a	AUB	Uterine mass	7.5	HE, BSO	48	
Kunz [32]	2007	38	G1, P0	asymptomatic	Intramural	12	Open TR	N/a	
Dede [33]	2008	37		AUB	Intramural	3.5	HE, LN sampling	N/a	
Anastasakis [34]	2008	28	G0, P0	AUB	Polypoid	N/a	Hsc TR	27	**Pregnancy** 6 mo after diagnosis
Berretta [35]	2009	26	G0, P0	AUB	Uterine mass		Hsc TR	N/a	
Stolnicu [36]	2009	71	N/a	AUB	Polypoid	2.5	HE, BSO	36	Adenosarcoma
Stolnicu [36]	2009	64	N/a	AUB	Polypoid	8	HE, BSO	60	Adenosarcoma, tamoxifen
Garuti [37]	2009	29	G0, P0	AUB	Submucosal	5	Hsc TR	13	
Aziz [38]	2009	62	N/a	PMB	Polypoid	2	TAH, BSO	N/a	Uneventful “yearly checkups”
Carta [39]	2010	74	N/a	AUB	Intramural	17	HE, BSO	8	
Giordano [40]	2010	26	G0, P0	AUB	Submucosal	2.2	Hsc TR	15	
Giordano [40]	2010	46	G2/P2	AUB	Polypoid	N/a	HE, BSO (finally)	N/a	Tamoxifen, breast cancer metastasis to the cervix
Abdullazde [41]	2010	46	N/a	AUB, Pain	Intramural	2	HE, BSO	24	Multiple leiomyomas
Abdullazde [41]	2010	30	N/a	AUB	“myoma”	2	“myomectomy”	N/a	
Abdullazde [41]	2010	42	N/a	AUB	Polypoid	1.5	HE	N/a	
Özer [42]	2013	38	G4/P3	AUB, pain	Intramural	18	TAH	N/a	Multiple leiomyomas
Abid [43]	2014	43	N/a	AUB	Polypoid and Intramural	1.5	D&C, HE, BSO	12	Multiple leiomyomas
Hashmi [44]	2014	48	N/a	AUB	Intramural	7	HE, BSO	N/a	
Ehdaivand [45]	2014	47	G0, P0	AUB	Intramural	N/a	Lsc HE, BSO, morcellation	24	Multiple leiomyomas
Gutierrez-Pecharroman [46]	2014	49			Polypoid	2	HE	18	Tamoxifen
Watrowski [47]	2015	22	G0, P0	AUB	Submucosal	2	Hsc TR	28	
Coelho [48]	2015	35	G0, P0	AUB	Submucosal	1.5	Hsc TR, followed by TAH	N/a	Multiple leiomyomas
Hermsen [49] (1)	2015	36	N/a	AUB	Submucosal	N/a	Hsc TR, Caesarean HE in 34 wop	24	**Pregnancy**
Hermsen [49] (2)	2015	68	N/a	AUB	Polypoid	1.5	D&C, TAH + BSO	6 (probably)	D&C misleading: ESS
Jeong [50]	2015	32	G0, P0	Infertility, AUB	Submucosal	3.6	Hsc TR, then Lsc HE after 5 mo	47	**Pregnancy**
Lin [51]	2015	37	G2, P2	AUB	Submucosal	5.7	Hsc TR, TAH	N/a	
Byun [52]	2015	56	G4, P2	AUB	uterine mass	2.2	HE, BSO	36	
Uçar [53]	2016	65	G6, P5	AUB	Intramural	8	TAH, BSO, PLND, PALND	12	
Gomes [54]	2016	53	N/a	AUB	Uterine mass	12	LASH, BSO, Cervix, OMx, parametrectomy, PLND	N/a	
Cetinkaya [55]	2016	52	G2, P2	AUB	Submucosal	2	HE, BSO, PLND, PALND	17	
De Franciscis [56]	2016	38	G0, P0	AUB	Polypoid	1	Hsc TR	60	**Pregnancy**
Cho [57]	2017	50	N/a	AUB		8.7	HE, BSO	N/a	
Schraag [58] (3)	2017	72	N/a	AUB	No macroscopic tumor	N/a	TAH, BSO	46	
Stefanovic [59]	2017	59	Multi-P	AUB	Polypoid	10	TAH, BSO	N/a	Two D&Cs (benign polyp) in 5 years prior to UTROSCT
Viau [60] (1)	2017	49	G1, P1	Pain	Intramural	1.8	HE, BSO	16	Multiple myomas of 8 cm
Sadeh [61]	2017	57	N/a	AUB	Polypoid	0.9	Lsc HE, BSO	36	
Varban [62]	2018	46	N/a	AUB	Intramural	7	HE, BSO	N/a	Subserosal myoma of 3.5 cm
Vilos [63] (1)	2018	52	G3, P3	AUB	Submucosal	1	Hsc TR; EMABL; LAVH, BSO	36	HE 9 mo after Dgn, no residues
Vilos [63] (2)	2018	47	G4, P3	AUB	Submucosal	2	Hsc TR, EMABL; LAVH, BS	12	
Fan [64]	2018	62	N/a	AUB	Uterine tumor	3.8	Extended HE, BSO, PLND, PALND	N/a	
Thakur [65]	2018	37	G1, P1	Infertility	Intramural	1.1	Hsc/Lsc TR	N/a	
Rozário Garcia [66]	2018	46	G1, P1	AUB	Prolapsed myoma	4	vaginal TR, then TAH, BSO	12	
Natarajan [67]	2018	58	Multi-P	AUB	Submucosal	4	TAH, BSO	N/a	
Zhang [68] (1)	2019	64	N/a	AUB	Uterine mass	10	HE, BSO	12	
Zhang [68] (2)	2019	33	G2, P1	AUB	Uterine mass	3.5	HE, BS	144	
Dubruc [69]	2019	56	N/a	AUB	Cervix	2.6	HE	4	
Segala [70]	2019	62	N/a	N/a	Intramural	7	TAH	10	Tamoxifen; multiple leiomyomas
Takeuchi [71]	2019	48	N/a	Abdominal fullness	Cervix	20	HE, BSO	N/a	Thoracic lymphadenopathy
Li [72]	2019	43	N/a	asymptomatic	Polypoid + Intramural	3.1	Lsc HE, BS (after HSc, D&C)	3	Endometriosis
Chiappa [73]	2019	28	G0, P0	AUB	Submucosal	5.5	Hsc TR	N/a	
Kim [74] (1)	2020	29	G1, P1	Pain	Subserosal	6.5	Lsc HE, BSO, PLND	3	
Kim [74] (2)	2020	49	G2, P2	AUB	Uterine mass	9	HE, BSO	12	
Nguyen [75]	2020	61	N/a	AUB	Polypoid	5.3	Extended Lsc HE, BSO, PLND	1	
Grither [76]	2020	69	N/a	AUB	Uterine mass	5.2	Robotic HE + BSO	8	
Sato [77]	2020	57	G2, P2	Pain	Intramural	2.5	HE, BSO, OMx, PLND, PALND	39	Sarcomatous features
Zhou [78]	2021	56	N/a	AUB	Pelvic mass	10	TAH + BSO	58	
Müller [79]	2021	18	G0, P0	AUB	Submucosal	4.5	Hsc TR (2×)	9	
Pereira [80]	2021	37	G0, P0	AUB	Submucosal	3.5	Hsc TR	20	
Pang [81] (1)	2022	46	N/a	AUB	Submucosal	4.5	Lsc HE	35	
Pang [81] (2)	2022	42	N/a	Pain	Intramural	5	Lsc HE	4	
Wang [82]	2022	42	N/a	AUB	Uterine mass	3.9	Lsc HE, BSO	N/a	
Xu [83]	2022	40	N/a	asymptomatic	Uterine mass	10	open TR	12	
Shibahara [84]	2022	77	G4/P2	AUB	Uterine mass	3	TAH + BSO	12	
Sahraoui [85]	2023	19	G0, P0	Pain	Cervix	3	cervical TR	24	
Yin [86]	2023	51		AUB	Submucosal	8.5	HE, BSO, LNE	12	GREB1-NCOA2 fusion
Ise [87]	2023	75		AUB		8			Myxoid features
Zhou [88]	2023	49	N/a	N/a	Intramural	14	HE, BSO	1	Increase in CA125
EL Hayek [89]	2023	58	G2, P2	Hemoperitoneum	Intramural	10	HE, BSO	N/a	Uterine rupture
Ferrara [90]	2023	73	G4, P2	AUB	Intramural	2.5	Lsc HE, BSO	12	
Present study	2023	31	G4, P4	Asymptomatic	Intramural	3	Lsc HE, BSO	N/a	
Present study	2023	58	Multi-P	AUB	Submucosal	1.5	Lsc HE, BSO	36	Flat submucosal area

AUB—abnormal uterine bleeding, N/a—not available, G—gravida, P—para, Hsc—hysteroscopic, Lsc—laparoscopic, TR—tumor resection, HE—hysterectomy, TAH—total abdominal HE, BSO—bilateral salpingo-oophorectomy, VH—vaginal HE, LND—lymphonodectomy, EMABL—endometrial ablation, and OMx—omentectomy; “uterine mass”—used when the exact localization within the uterus was not reported.

**Table 4:** Aggressive cases (extrauterine spread or metastasis at the first diagnosis or recurrence).

First Author	Year	Age	Site (Extrauterine Extension)	Size, cm	Primary Treatment	Recurrence and PFS	Total FU	Last Status
Kantelip [91]	1986	86	Intramural, left ovary, two epiploic nodules	10	TAH, BSO, partial cystectomy, epiploic resection	No recurrence	60 mo	NED
Malfetano [92]	1989	18	Intramural	5	TR, followed by TAH, PALND, Omx	Sigmoid, mesentery, ovary (nodules up to 6 cm), abdominal wall nodules 1–2 cm, PFS 72	N/a	Tumor initially diagnosed as LG-ESS, recurrence as UTROSCT, G0, P0
Di Vagno [93]	1996		Pelvic tumor, lung metastasis, carcinomatosis (35th gw)		Caesarean HE, debulking, two CHT regimens (nonresponsive)	PD	9 mo	DOD, 9 mo after dgn
Biermann [94]	2007	68	Intramural	4.5	HE	10 cm, small bowel, PFS 48 mo	48 mo	2 benign gastrointestinal stromal tumors
O’Meara [95]	2009	35	Intramural	9.9	HE	Retropubic mass with bladder invasion, 8.3 cm, PFS 3 ys	48 mo	NED 1 ys after secondary treatement with surgery and CHx, galactorrhea and hyperprolactinemia (at first and second dgn)
Blinman [96]	2009	49		6.5	HE	8 cm retroperitoneal mass, PFS 11 ys		Response to second-line anastrazole, lost for follow-up 10 ys after FD
Macak [97]	2014	53	Uterine mass	1.5	HE, BSO, PALND	No recurrence	10 mo	NED
Umeda [98] (1)	2014	38	Submucosal	4.5	HE, BSO, PLND	No recurrence	11 mo	NED
Umeda [98] (2)	2014	57	Submucosal	6.4	HE, BSO	No recurrence	8 ys	NED
Endo [99]	2015	62	N/a	N/a	HE	Pelvic recurrence, 14 cm, 23 ys after HE (PFS 276 mo)	24.5 ys	SD (recurrent tumor not completely resected)
Kuznicki [100]	2017	49	Ovary, omental cake	6	Neoadjuvant CHx, optimal cytoreduction	PD, death 15 mo after dgn	15 mo	DOD (CA125: 2210 U/mL)
Schraag [58] (1)	2017	24	Submucosal	N/a	Hsc TR, followed by re-Hsc, followed by open TR	Uterus, PFS 9 mo	65 mo	NED, 56 mo from last surgery. False positive MRI (8 mm nodule) after 3 mo
Schraag [58]	2017	28	Myoma	10	open TR	Pelvis, PFS 20 mo	55 mo	Tumor rupture during first surgery; **pregnancy** after second surgery
Viau [60]	2017	43	Double tumor: pedunculated uterine mass (13 cm), myometrial mass (5.5 cm); peritoneum		TAH, BSO, debulking, CHTx (bleomycin, etoposide, cisplatin).	Pelvic tumor 5.5 cm, PFS 40 mo	64 mo	NED, 2 years after second surgery Tumor rupture during first surgery
Kondo [101]	2017	69	Uterine mass		TAH, BSO	Lung, PFS 26 mo		NED
Cömert [102]	2018	61	Pelvic mass	7	TAH, BSO	Pelvic mass, spleen, omentum, PFS 60 mo	83 mo	NED, 7 mo after last surgery
Marrucci [103]	2019	54	Uterine mass	9	HE, BSO	vaginal vault, PFS 50 mo	74 mo	NED 24 mo after recurrence, Coexistence with multiple leiomyomas
Bennett [104] (1)	2020	32	Intramural	N/a	HE	(1) Pelvic sidewall, PFS 7 ys, and (2) second subtotal debulking, PFS 11 mo	8 ys	AWD (Second subtotal debulking 11 mo later)
Bennett [104] (2)	2020	54	Intramural (multiple tumors 1.5–6.5 cm)	1.5–6.5	LASH, followed by trachelectomy	Pelvis, PFS 9 ys, debulking, CHx	10 ys	NED
Bennett [104] (3)	2020	30	N/a	N/a	HE	Omentum, PFS 32 ys	6 ys	PD (2 further recurrences, 2 and 4 years later)
Chang [105]	2020	57	Intramural	10	TAH, BSO	Pelvic mass, PFS 30 mo	35 mo	GREB1-NCO2 rearranged
Sh Hassan [106]	2020	41	Intramural	N/a	TAH	Vaginal vault, PFS “few weeks”	24 mo	NED
Dimitriadis [107]	2020	46	Uterine mass	11	TAH	Intraabdominal recurrence, PFS 2 ys	2 ys	N/a (report at the time of relapse)
Dondi [108]	2021	24	Submucosal	3	Hsc TR	Uterus, PFS 20 mo	30 mo	NED after secondary Lsc HE
Devereaux [109]	2021	42	Intramural	8.8	TR with morcellation; at recurrence: TAH, BSO, debulking	Uterus, PFS 6 mo	6 mo	Lost for FU after second surgery
Chen [110]	2021	33	Uterus, pelvic lymph nodes	N/a	Radical HE, BSO, PLND, CHx, RTx	Retroperitoneal mass in the upper abdomen 10 × 7 cm, PFS 14 ys	14 ys	Initially diagnosed as LG-ESS with pelvic LN metastases
Wei [111]	2021	46	Uterus	11	TAH, BS	20cm pelvic tumor adherent to intestine, PFS 53 mo.	62 mo	DOD (9 mo after relapse); Ki67 25%, p53 positive; D&C 2 mo earlier: normal
Massa [112]	2022	56	Intramural, among multiple myomas	N/a	HE	10 peritneal nodules up to 8 cm, PFS 7 ys	17 ys	DOD, 10 CHTx, antibody and hormonal therapies

ys—years, mo—months, N/a—not available, HE—hysterectomy, TAH—total abdominal hysterectomy, LASH—laparoscopic supracervical hysterectomy, LAVH—laparoscopically assisted vaginal hysterectomy, BSO—bilateral salpingo-oophorectomy, BS—bilateral salpingectomy, Hsc—hysteroscopic, Lsc—laparoscopic, TR—tumor resection, OMx—omentectomy, Rx—radiotherapy, CHx—chemotherapy, LN—lymph nodes, LND—lymphonodectomy, PFS—progression-free survival, DOD—dead of disease, NED—no evidence of disease, PD—progressive disease, and FU—follow-up.

**Table 5:** Case series with clinical data (in ≥ 3 categories).

First Author	Year	No.	Age	Symptoms	Localization	Size (cm)	Primary Treatment	Recurrence, PFS	Total FU	Outcome by Last-Seen	Comments
Clement [4]	1976	14	44 (type 1, n = 6) 49 (type 2, n = 8)	AUB (n = 9) Pelvic discomfort (n = 2) Asymptomatic (n = 3)	Intracavitary (n = 1 type 1, n = 3 type 2) Submucous (n = 1, type 1) Intramural (n = 3, type 1, n = 2, type 2) Subserosal (n = 3 type 2) Extrauterine spread (n = 1, type 1)	2–15	Type 1: TAH, BSO (n = 5); VH (n = 1) Type 2: TAH, BSO (n = 7), TAH (n = 1)	Type1: Yes (n = 3): (a) PFS 12 ys, irradiation; (b) PFS 2 ys, lung metastases, no therapy; (c) PFS 2 ys, CHx-, Rx; Type 2: No	Type 1: 22 mo–15 ys Type 2: 4 mo–7 ys	Type 1: DOD: n = 2, NED: n = 1 after relapse; NED: n = 2 w/o relapse; lost for FU: n = 1 Type 2: NED (all)	
Baker [113]	1999	15	50 (30–78), (type 1, n = 10) 51 (34–77) (type 2, n = 5)	AUB (n = 5) Pelvic mass (n = 8); Asymptomatic (n = 2)	Intramural or polypoid (no details)	N/a	N/a	N/a	N/a	N/a	
Irving [114]	2006	8	42 (19–69)	AUB (n = 8)	Intracavitary polyp (n = 3) Intramural (n = 4) n/a (n = 1)	3.5–14	TAH, BSO (n = 2); TAH, BSO, Rx (n = 1), TAH, BSO, CHx (n = 1), HE (n = 4)	No (n = 7) Yes (n = 1; Lung, bone, PFS N/a, death 10 mo after dgn	10–62 mo	NED (n = 7) DOD (n = 1, type 1)	
Rollins [115]	2007	37	47 (21–66)	N/a	Submucosal (“majority”)	2.9 (0.7–17)	N/a	N/a	N/a	N/a	
Hurrel [6]	2007	4	43, 51, 73, 84		Intramural (n = 3) Pedunculated/subserosal (n = 1)	0.8–19.5	HE (4 times), BSO (2×), ULSO (1×)	N/a	N/a		
Nogales [116]	2009	6	65 (42–76)	AUB (n = 4) Asymptomatic (n = 2)	Polypoid (n = 4) Intramural (n = 2)	0.7–8	TAH, BSO (n = 5) Rx (n = 1)	No	1–15 ys	NED	Pelvic endometriosis: n = 1 tamoxifen: n = 1
Staats [117]	2009	24	51 (29–84)	N/a	Endocervical polyp (n = 1); Submucous (n = 7); Intramural (n = 10); Subserosal (n = 2)	6.6 (2–22)	N/a	N/a	N/a	N/a	Ultrastructural study; cases from the Collection of Scully
de Leval [118]	2010	12	50 (29–59)	AUB (n = 5) Asymptomatic (n = 4) N/a (n = 3)	Intramural (n = 5), Polyp or submucous (n = 4), subserosal (n = 1); N/a (n = 2)	5.5 (3–10)	TAH, BSO (n = 6), TAH (n = 1), VH (n = 1); D&C (n = 1), N/a (n = 3)	N/a	N/a	N/a	Ultrastructural study; cases from the Collection of Scully
Bakula-Zalewska [119]	2014	6	50,25, 51,63, 24,62	N/a	Uterine mass	3–24	LASH + BSO (4 times), HSC TR (Case 2 and 5), adjuvant gestagene (4 times, nos)	No	3–14.5 ys	NED	
Liu [120]	2015	5	45 (35–50)	AUB (n = 4) Asymptomatic (n = 2)	Intramural (n = 3); Polypoid/submucous (n = 2); Protruding mass (n = 1)	5.6 (3–10.2)	TAH (n = 3); VH (n = 1); TAH, BSO (n = 1); TR (n = 1)	Yes (n = 2) No (n = 4)	3 mo–7 ys	NED (n = 4) AWD (n = 1) N/a (n = 1)	CIN (n = 1); 1 recurrence after Hsc TR, one after TAH.
Stewart [121]	2016	6	60 (42–67)	AUB (n = 6)	Intramural (n = 5) Endocervial (n = 1)	4.6 (1–10)	N/a	No	65.5 (39–96) mo	NED	
Moore [122]	2017	34	52 (12–86)	N/a	Uterine mass Metastasis (n = 1)	6.1 (0.4–19.5)	HE (n = 30) TR (n = 2) CHx and Rx (n = 1, metastatic disease at first diagnosis)	No (n = 26) Yes (n = 7; LN, pelvis, lung, bone, liver; PFSs 11–78 mo)	39 (6–135) mo	DOD (n = 3; 12, 23 and 23 after diagnosis); PD (n = 5) NED (n = 26)	
Croce [123]	2019	12	70 (n = 1) N/a (n = 11)	Pelvic mass (n = 1) N/a (n = 11)	N/a	10 (n = 1) N/a (n = 11)	TAH, BSO; posterior exenteration for recurrence (n = 1); N/a (n = 11)	Yes (n = 1, PFS 17 mo, pelvis, lung) N/a (n = 11)	29	N/a	11/12 cases only as ultrastructural study
Dickson [124]	2019	4	53 (38–68)	N/a	Intramural (n = 3) Polypoid (n = 1)	2.4 (0.7–3.3)	HE (n = 3), D&C (n = 1)	N/a	N/a	N/a	Adenomyosis
Goebel [124]	2020	26	49.6 (20–74)	N/a	Polypoid (n = 4) Intramural (n =11) (data available for 15 tumors)	5.1 (0.5–15)	HE (n = 17), TR (n = 3), D&C (n = 6) (numbers refer to the specimen source) *	No (n = 10) Yes (n = 1, pelvis, DFS 66 mo.) N/a (n = 16)	94.4 (1–319) mo	NED (n = 11)	
Kaur [126]	2020	6	42, 43, 46, 47, 49, 50.	AUB (n = 6)	Intramural (n = 5) N/a (n = 1)	1–9.3	TAH, BSO (n = 4); TAH, BSO, CHTx (n = 1); radical HE Type 3, BSO (n = 1)	Yes (n = 1, PFS 7 mo), No (n = 5)	4 weeks–2 ys	NED (n = 4) N/a (n = 1)	Tamoxifen: n = 1
Carbone [127]	2021	10	48.5 (30–69)	AUB (n = 8) Miscarriage (n = 1) Asymptomatic (n = 1)	Intramural (n = 10)	2 (0.2–8)	HE, BSO (n = 4), HE, BSO, LNE (n = 3), LASH, BSO, Hsc TR (n = 1), open TR (n = 1)	No	25 (3–97) mo	NED	Both conservatively treated patients became **pregnant**
Ye [128]	2022	5	53 (39–65)	AUB (n = 5)	Polypoid (n = 3) Intramural (n = 2)	1.5–5	HE, BSO (n = 4), HSC TR (n = 1)	No	20 (4–72) mo	NED	
Boyraz [129]	2023	75	53 (21–84)	AUB (n = 35) Pelvic pain (n = 6) Asymptomatic (n = 16) N/a (n = 18)	Intramyometrial (n = 38) Submucosal (n = 34) cervical (n = 3) Lung metastasis (n = 1)	3.5 (0.6–20)	HE (n = 18), HE, BSO (n = 53), TR (n = 4)	Yes (n = 4); 1. peritoneum, PFS 60 mo; 2. peritoneum, PFS 144 mo; 3. peritoneum, PFS 60 mo; 4. brain and femur, PFS 30 and 48.	64 (22–192) mo	NED (n = 53), AWD (n = 3), DOD (n = 2)	
Xiong [130]	2023	19	42.8 (19–58)	N/a	N/a	4.1 (1.5–15)	HE (n = 11) No treatment (n = 1) N/a (n = 7)	No (n = 13), Yes (n = 6): 1. Peritoneum, PFS 99 mo; 2. pelvis, colon, PFS 2 mo, death; 3. Site n/a, PFS 54 mo; 4. lung, pelvis; PFS 13 mo; 5. pelvis, colon; 189 mo; 6. lung, PFS 14 mo)	40.9 (1.2–195.3) mo	NED (n = 18, incl. n = 5 after recurrence) DOD (n = 1)	
Lu [131]	2023	18	45 (27–60)	AUB, pelvic mass (n not indicated)			HE (n = 3) HE, BSO (n = 8) Hsc TR (n = 5) Lsc TR (n = 2)				
Bi [132]	2023	23	43 (23–65)	N/a	Intramyometrial (n = 14) Polypoid/submucosal (n = 7) Protuberant mass (n = 2)	5.4 (1–15)	TAH (n = 4) TAH, BSO (n = 13) TAH, BSO, LND (n = 2); TR (myomectomy) (n = 2); TR (polypectomy) (n = 2)	Yes (n = 8) No (n = 15)	8–177 mo	NED (n = 21) DOD (n = 2)	
Bini [133]	2023	4		N/a	Metastatic tumors	N/a	N/a	Yes (n = 4)	13.5 (6–34) ys	DOD (n = 3) NED (n = 1)	
Qijun [134]	2023	17	47 (19–67)	AUB (n = 15) Asymptomatic (n = 2)	Intramural (n = 10) Submucosal (n = 7)	4.6 (0.6–14.7)	TAH or Lsc HE (n = 13) Hsc TR (n = 4) CHx (n = 1)	Yes (n = 3) plevis/abdomen, PFS 16 and 17 mo; lung PFS 12 mo; No (n = 14)	20.2 (1–68) mo	NED (n = 14) N/a (n = 3)	

* (mean or median, range), ys—years, mo—months, Rx—radiotherapy, CHx—chemotherapy, AUB—abnormal uterine bleeding, N/a—not available, HE—hysterectomy, TAH—total abdominal hysterectomy, LAVH—laparoscopically assisted vaginal hysterectomy, BSO—bilateral salpingo-oophorectomy, Hsc—hysteroscopic, Lsc—laparoscopic, TR—tumor resection, VH—vaginal hysterectomy, LN—lymph nodes, LND—lymphonodectomy, PFS—progression-free survival, DOD—dead of disease, NED—no evidence of disease, and AWD—alive with disease.

**Table 6:** Common partner genes related to GREB1 and ESR1 rearrangement in UTROSCT.

Gene	Encoded Protein	Function	Reference
GREB1	Growth Regulation by Estrogen in Breast Cancer 1	Transcriptionally driven by estrogen-bound ER, important in the estrogen/ER signaling pathway. GREB1-rearranged UTROSCT may be more aggressive	[76,86,104,105,110,125,132,134,141]
ESR 1	Estrogen Receptor 1	Ligand-dependent transcription factor involved in sexual development, reproduction, and bone formation. ESR1-rearranged UTROSCT may be resistant to estrogen blockade due to loss of the ER ligand-binding domain	[76,104,105,132,133,134,141]
NCOA1–3	Nuclear Receptor Coactivator 1–3	Enhance the activity of nuclear hormone receptors and mediate transcriptional effects of steroid/sex-hormone receptor pathways. Fusions involving NCOA genes have oncogenic potential when dysregulated	[76,104,105,124,125,131,134,141]
CTNNB1	β-Catenin	Key in Wnt/β-catenin signaling pathway, coactivator for TCF/LEF, involved in transcription initiation and chromatin remodeling.	[105,123]
NR4A3	Nuclear Receptor Subfamily 4 Group A Member 3	Transcriptional activator for the steroid/thyroid hormone nuclear receptor family, regulating proliferation, survival, and differentiation.	[105]
GTF2A1	General Transcription Factor IIA, subunit 1	Component of the RNA polymerase II transcription-initiation complex, interacting with steroid hormone receptors, including ERα, to facilitate transcription initiation.	[109]
CITED2	CBP/p300 interacting transactivator with Glu/Asp-rich carboxyl-terminal domain 2	Transcriptional co-activator that modulates interactions between DNA-binding proteins and histone modifying enzymes, influencing the transcription of genes involved in embryonic development or cellular response to hypoxia.	[134]

## Data Availability

The data presented in this study are available on request from M.P. and R.W.

## Appendix A

**Table A1:** Patients with pregnancy concurrent to or following UTROSCT.

First Author, Year	Age	Symptoms	Size (cm)	Treatment	Infiltrative Margins	Conception	Time from dgn (mo)	Delivery Mode	Recurrence	HE after Childbearing	Total FU, mo
Di Vagno, 1995 [93]	N/a	Hemoperitoneum at 35 weeks	5	Caesarean HE	Yes	SpoCo, concurrent with UTROSCT	0	CS, 35 weeks	PD	Yes	9
Hillard, 2004 [25]	32	Infertility; AUB	N/a	Lsc TR		SpoCo	15	N/a	No		15
Anastasakis, 2008 [34]	28	AUB	N/a	Hsc TR	No	SpoCo	6	VD	No	No	27
Hermsen, 2015 (Case 1) [49]	36	AUB	N/a	Hsc TR	Yes	SpoCo, concurrent with UTROSCT	0	CS (with HE), 34 weeks	No	Caesarean HE at 34 weeks (no reisdual tumor)	24
Jeong, 2015 [50]	32	Infertility, AUB	3	Hsc TR	Yes	In vitro fertilization	3	CS, 36 weeks, 3070g	Yes	Lsc HE (residual tumor on specimen)	47
De Franciscis, 2016 [56]	38	Infertility, AUB	1	Hsc TR	Yes	SpoCo	2	CS, 39 weeks	No	No	60
Schraag, 2017(Case 2) [58]	28	Pelvic pain	10	Open TR	No	SpoCo (after second surgery for residual tumor)	19	CS (with HE) at 39 weeks	Yes (20 mo after CS)	Caesarean HE at 39 weeks	55
Carbone, 2021 (Case 1) [127]	25	AUB	0.2	D&C (for miscarriage) followed by Hsc TR	N/a	1st: SpoCo concurrent with UTROSCT, 2nd: SpoCo	24	VD, 39 weeks, 3590 g	No	No	96
Carbone, 2021 (Case 2) [127]	30	AUB	4	open TR	No	SpoCo	13	CS, 38 weeks, 3080 g	No	No	16

ys—years, mo—months, AUB—abnormal uterine bleeding, N/a—not available, HE—hysterectomy, Hsc—hysteroscopic, Lsc—laparoscopic, TR—tumor resection, and SpoCo—spontaneous conception.
